# Leveraging win time endpoint and combining moderators for personalized treatment recommendation in time-to-event clinical trials

**DOI:** 10.1186/s12874-026-02960-8

**Published:** 2026-08-15

**Authors:** Stephanie Pan, Janice Weinberg, Sara Lodi, Prasad Patil, Michael P. LaValley

**Affiliations:** Department of Biostatistics, Boston University School of Public Health, Boston, MA 02118 USA

**Keywords:** Effect modification, Composite moderator, Randomized trials, Personalized medicine

## Abstract

**Background:**

There has been increasing interest in randomized trials on the discovery and identification of treatment moderators on an outcome. This reflects the stratified medicine paradigm, which moves beyond average treatment effects to focus on patient subgroups with heterogeneous responses. Baseline biomarkers and clinical variables are commonly evaluated as candidate moderators and standard subgroup analyses are typically used to identify single moderators. Recent research has sought to improve the detection of effect modification by optimally combining multiple moderators into a single composite measure. This composite moderator can offer greater power and sensitivity to detect effect modification over any single moderator alone and can be used in medical applications to derive personalized treatment recommendations (PTRs).

**Methods:**

Kraemer applied this concept to continuous outcomes, and we extended this approach to time-to-event settings using the expected win time against reference (EWTR), an estimand that measures time spent in better or worse clinical states. We compared the composite moderator with single moderators and explored its utility by evaluating whether there were improved treatment recommendations in simulations that varied (1) effect modification scenarios (quantitative vs qualitative), (2) interaction-to-main-effect ratio magnitudes, and (3) configurations of the moderator correlations with the endpoint. We illustrated this approach using randomized trial data investigating two management strategies for participants with atrial fibrillation.

**Results:**

Simulations demonstrated that in the presence of strong effect modification, the composite moderator matched or outperformed the best single moderator. However, its advantage was attenuated by weak interactions, correlated moderators, or inclusion of a null endpoint.

**Conclusions:**

We extended Kraemer’s composite-moderator framework to time-to-event outcomes using EWTR. The composite moderator may serve as a pragmatic tool for detecting effect modification and informing personalized treatment recommendations, particularly when effect modification is moderate to strong.

**Supplementary Information:**

The online version contains supplementary material available at https://doi.org/10.1186/s12874-026-02960-8.

## Introduction

In randomized trials characterized by treatment effect heterogeneity, attention shifts from the average treatment effect to identifying subgroups with favorable responses and those for whom treatment may be less effective or even potentially harmful [1–3]. When treatment benefits differ across a population, baseline variables can be leveraged to construct a personalized treatment recommendation (PTR) algorithm that aims to improve expected clinical outcomes by selecting the preferred treatment over the alternative [1, 4, 5]. To stratify a population into subgroups, candidate variables should function as *moderators *(or treatment effect modifiers) rather than as prognostic markers, which predict the outcome independent of treatment. Under the MacArthur criteria, a moderator is measured prior to treatment randomization, is independent of treatment assignment, and predicts treatment response [6–8]. In contrast, a mediator is a post-treatment variable on the causal pathway between the treatment and outcome, and the mediator may be correlated with treatment [6, 7, 9]. Mediators provide insight into the treatment processes, such as adjustments to the treatment regimen, while the focus of this study is to use moderators to stratify populations into subgroups for improved targeting of treatments [6, 7]. Thus, a key aim of this work is to evaluate the strength and impact of moderators to guide personalized treatment decisions.

There are two broad patterns of effect modification (or interactions with treatment) that are clinically relevant. In quantitative interactions the treatment effects may differ in magnitude across moderator levels, but the direction is uniform. In qualitative interactions, the direction of the treatment effect reverses across moderator levels, implying that treatment is favorable for some but may be harmful for others. While qualitative interactions are less common in real-world clinical trial settings, relative to quantitative interactions, their presence would raise important considerations for the interpretation of trial results and treatment decisions [10–12].

Although the conventional method is to examine single biomarkers or other potential moderators with treatment for effect modification, in some therapeutic areas individual biomarkers or moderators may be insufficient to delineate clinically meaningful subgroups. Kraemer’s approach addresses this by estimating a composite moderator as a weighted combination of multiple moderators, with weights chosen to maximize the association between pairwise outcome differences and average moderator values from all treated-control pairs [13, 14]. The resulting composite moderator can improve the detection of effect modification and yield better treatment recommendations than a single moderator, which often may have modest impact.

Most applications of moderator assessment have focused on continuous outcomes, typically using linear regression models on an additive scale for interaction testing [15]. Some authors argue that additive effects provide stronger evidence of underlying interactions than multiplicative effects [16–18]. However, in cardiovascular and other therapeutic areas of clinical trials, time-to-event endpoints analyzed with Cox regression models, which utilize a multiplicative scale, predominate [15, 19]. To reflect the clinical experience of a participant through time and the flexibility of incorporating composite hierarchical events, win time methods are variants of the win ratio and summarize overall treatment benefit (or harm) based on how long participants spend in better (or worse) clinical states [20]. We focus on the *expected win time against reference *(EWTR), which compares each participant’s clinical state through time to a reference clinical state distribution [20]. The EWTR can be compared between treatment groups via a standard t-test or linear model. Unlike a composite time-to-event endpoint, which considers only the first event, or the win ratio, which emphasizes the first high-priority event, the EWTR accounts for transitions through ordered clinical states and can provide a more comprehensive summary of clinical experience [20].

We extended Kraemer’s composite moderator framework for time-to-event outcomes by using EWTR as the outcome in modeling treatment-moderator interactions and adapted this approach to construct a PTR. We then evaluated whether the composite moderator improves personalization performance versus single moderators under: (i) qualitative and quantitative interaction patterns, (ii) a range of interaction-to-main-effect ratio magnitudes, and (iii) other conditions such as larger sample sizes, correlation among moderators, number of moderators, and clinical endpoint considerations. The “Background” section reviews the derivation of the composite moderator and computation of EWTR. The “Simulation” and “Simulation results” sections present the simulation design, construction of the PTR, and results. We applied this extended approach to clinical trial data comparing two management strategies for participants with atrial fibrillation in the “Application” section. The “Discussion” section concludes the article.

## Background

### Kraemer approach for creating a composite moderator

Kraemer’s approach estimates an overall effect modification by constructing a composite moderator (M*) as a weighted sum of individual moderators [13]. First, it uses a parametric model fitted to all pairwise combinations of treated and control paired participants:

1 $$\begin{aligned}\Delta Y=&{Y}^{(1)}-{Y}^{(0)}={w}_{1}*{\overline{E} }_{1}+{w}_{2}*{\overline{E} }_{2}\\&+\dots +{w}_{M}*{\overline{E} }_{M}\end{aligned}$$

2 $${\overline{E} }_{m}=\frac{{E}_{m}^{(1)}+{E}_{m}^{(0)}}{2},m=1,\dots , M$$

where ${Y}^{(1)}$ and ${Y}^{(0)}$ are the outcomes for the treated and control participants in a paired comparison, respectively, and *Δ Y* is their difference; *m=1, … , M* indexes the *M* single moderators; ${E}_{m}$ is the m^th^ baseline moderator, with ${E}_{m}^{(1)}$ and ${E}_{m}^{(0)}$ denoting the values of moderator *m* for the treated and control participants, respectively; ${\overline{E} }_{m}$ is the paired average for moderator *m*; and ${w}_{m}$ are the moderator-specific weights estimated by regressing *Δ Y* on the set ${\{{\overline{E} }_{m}\}}_{m=1}^{M}$for all treated and control pairs [13]. Moderators can be of any data type. Binary variables should be effect-coded and continuous variables should be standardized. Categorical moderators should be recoded into dummy variables and ordinal moderators can be treated as numeric or categorical, depending on the context. The goal is to maximize the correlation between pairwise outcome differences (*Δ Y*), which captures the treatment effect within each pair, and the average moderator values (${\overline{E} }_{m}$) across pairs. The average moderator represents the baseline trait of the pairs and how this influences the magnitude of the treatment effect. Once weights ${w}_{m}$ are estimated from the pair-level regression, the composite moderator (M*) for each individual *i* is computed as:

3 $${M}_{i}^{*} =\sum\:_{m=1}^{M}{w}_{m}{E}_{im}$$

which can amplify the overall effect modification detection and support a personalized treatment selection strategy [4, 13, 14]. In the “Simulation” section, we discuss how this composite moderator can be used to construct a PTR, and in the “Simulation results” section, we examine settings in which the composite moderator may offer advantages over a single moderator.

### Expected win time against reference

Troendle et al. recently proposed several methods on the use of *win time *for capturing the overall treatment benefit from an ordered composite endpoint [20]. The appeal of utilizing win time is to better reflect the participant clinical experience, measured in time, as they progress from less severe to more severe clinical events. We focus on one of these methods, the *expected win time against reference* (EWTR), which first estimates a distribution of clinical states from baseline (${t}_{0}$=0) through the time of the last participant followed under a reference (or control) group (${\tau}_{r=0}$). Each participant’s clinical state is compared to this reference clinical state distribution. The EWTR can then be compared between treatment groups via a standard t-test or linear model. Briefly, the method is as follows and details can be found in the original publication.

To estimate the reference group’s clinical state distribution through time we used the reference group of the trial with the extended Markov model, as suggested by the authors [20]. Let ${t}_{0}< {t}_{1}<\dots < {t}_{K}$= ${T}_{max}$ be the ordered, distinct event or censoring times observed in the reference group. We assume all participants begin at the baseline state. When a participant experiences an event and transitions to another state, there is a reallocation of state probabilities from the previous state to the new state. Participants who are censored at ${t}_{k}$ are removed from the risk set at ${t}_{k}$, and there is no reallocation in the state probability distribution. Assuming a baseline and two clinical states (*s* = 0, 1, 2), the estimated clinical state distribution for the reference group is: $\widehat{{p}_{0}}\left(t\right), \widehat{{p}_{1}}\left(t\right)$, $\widehat{{p}_{2}}\left(t\right)$.

Next, each participant *i* is compared to this reference group’s clinical state distribution until the effective common follow-up time, ${\tau}_{i}\equiv \mathrm{m}\mathrm{i}\mathrm{n}({\tau}_{r},{\tau}_{i}^{*})$, where ${\tau}_{i}^{*}$ is the censoring time for participant *i* or *∞* if the participant *i* dies. To compare each participant i to the distribution, the following state-specific win components [${wc}_{i0}\left(t\right), {wc}_{i1}\left(t\right), {wc}_{i2}\left(t\right)$] are assigned: *+1* if participant *i* is in a *better* state at time ${t}_{k}$ than state *s*, *-1* if the participant *i* is in a *worse* state at time ${t}_{k}$ than state *s*, and 0 otherwise for *s* = 0, 1, 2. The estimated EWTR for each participant *i* is the time in a better clinical state compared to the reference group over the average effective common follow-up time [20]:

4 $$\widehat{{EWTR}_{i}}= {\int}_{{t}_{0}}^{{\tau}_{i}}\{{wc}_{i0}\left(t\right)\widehat{{p}_{0}}\left(t\right)+{wc}_{i1}\left(t\right)\widehat{{p}_{1}}\left(t\right)+ {wc}_{i2}\left(t\right) \widehat{{p}_{2}}\left(t\right)\}.$$

It is worth noting that when a participant dies, the EWTR method takes into account a longer period of time following death than other win time methods. This method places greater weight on early death when there is a long effective common follow-up time and it can be sensitive to treatment effect on death [20].

### Personalized treatment recommendation

A personalized treatment recommendation (PTR) refers to an algorithm that utilizes a participant’s measured baseline characteristics to recommend one treatment strategy over another [1, 3, 4, 21–23]. Here, “treatment strategy” refers specifically to the assigned intervention in a two-arm randomized trial and does not refer to dynamic treatment regimes that adapt over time. The intention of this algorithm is for clinical use and to improve the expected clinical outcome, assuming a population with heterogeneous subgroups as opposed to assigning treatment based on a population-average response. Within stratified medicine, the PTR framework addresses heterogeneity of treatment effect by identifying subgroups for whom a given treatment strategy is expected to yield greater benefit [1, 2, 4].

The PTR is defined to select the treatment that maximizes the EWTR for a given set of baseline characteristics. As such, at the individual level, the objective is to maximize expected time spent in more favorable clinical states, as quantified by EWTR. At the population level, the target estimand is the mean EWTR under the PTR strategy compared with non-personalized strategies (e.g., assigning all participants to a single treatment). “Gains in personalization” are defined as the expected increase in EWTR under the PTR strategy relative to these non-personalized strategies.

In our study, we first summarize each participant’s expected treatment benefit with EWTR on two ordered time-to-event endpoints. Next, we utilize the composite moderator M* derived from the weighted sum of single baseline moderators via Kraemer’s method [13] to develop the PTR strategy, or the individual-level decision rule. We regress the EWTR outcome on a model with treatment (*Z*), composite moderator, and the interaction term between treatment and the composite moderator. These parameters are used to compute PTR:

5 $$PTR= I\left\{\left({\beta}_{0}^{*}+{\beta}_{1}^{*}{M}^{*}\right)>0\right\}$$

where ${\beta}_{0}^{*}$ is the average effect of the treatment, ${\beta}_{1}^{*}$ is the average effect modification, ${M}^{*}$ is the composite moderator, and *I()*is the indicator function for recommending the treatment strategy over the control strategy [4]. In the “Personalized treatment recommendation” and “Performance measures” sections, we detail the validation process and specific metrics used across our simulations to evaluate the utility of this algorithm under PTR and non-personalized strategies. In the “Application” section, we apply the algorithm to data from a randomized controlled trial of participants with atrial fibrillation and estimate the mean outcome under a PTR strategy [4]:

6 $$\begin{aligned}\mu \left\{PTR\right\}= &\frac{1}{n}\sum^{n}(\frac{\left(Z+\frac{1}{2})\ (PTR+\frac{1}{2}\right)}{\pi }EWTR\\&+\frac{(\frac{1}{2}-Z)\ (\frac{1}{2}-PTR)}{1-\pi }EWTR)\end{aligned}$$

The expected outcome under a PTR strategy is the average of the observed outcome for those receiving the recommended treatments, weighted by the probability of being randomized to that group (*π*). Note that treatment, *Z*, is effect-coded −1/2 and 1/2 for control and treatment for clearer interpretability [13, 24, 25]. Under non-personalized strategies, the expected outcome under treatment is [4]:

7 $$\mu \left\{Treatment\right\}=\frac{1}{n}\sum^{n}(\frac{Z+1/2}{\pi }EWTR)$$

and for control:

8 $$\mu \left\{Control\right\}=\frac{1}{n}\sum^{n}(\frac{1/2-Z}{1-\pi }EWTR).$$

After delineating participants into recommendation strata, one can explore a range of borderline composite moderator M* values to characterize participants at the decision boundary who may benefit from additional clinical decision-making.

## Simulation

### Data generating mechanism and scenarios

Simulations were conducted to explore the performance of the Kraemer [13] composite moderator framework under various scenarios. The configuration, which included five effect moderators and two time-to-event outcomes, was loosely based on the randomized trial example presented later. The setting is a two-armed randomized clinical trial comparing treatment to a standard of care, with treatment randomly assigned independently of baseline moderators. The simulation assumes a standard parallel-group randomized design with a fixed sample size. We simulated 1000 trials of size 1000 and 2000 with 1:1 randomization, with participants independently randomized at baseline.

#### Time-to-event outcomes

We assume two clinical events, one is non-fatal (e.g., non-fatal adverse event, hospitalization) and the other is death; the clinical ordering of events is non-fatal event < death. To simulate the two event times, we allow the times to be correlated within individuals by including an additional covariate *U*, an unobserved subject-specific frailty that follows a uniform distribution ($-\frac{\Psi }{2},\frac{\Psi }{2}$) that will be common across the two clinical event models. We simulate data according to the methods of Beyersmann et al. and Troendle et al. by using a proportional cause-specific hazard (CSH) model for each of the two clinical events ($s=1\ or\ 2$) and for censoring (*s=3*) [20, 26]. The CSHs ${\lambda}_{s=1}\left(t\right)$ and ${\lambda}_{s=2}\left(t\right)$ for the non-fatal event and death are given by:

9 $$\begin{aligned}{\lambda}_{s=1}\left(t\right)=&exp[{\alpha}_{1}+{\beta}_{1}*Z+{\gamma}_{1}*U\\&+\sum\:_{m=1}^{5}({\delta}_{1m}* {E}_{m}+ {\delta}_{1m}^{*}* {E}_{m}*Z)]\end{aligned}$$

10 $$\begin{aligned}{\lambda}_{s=2}\left(t\right)=&exp[{\alpha}_{2}+{\beta}_{2}*Z+{\gamma}_{2}*U\\&+\sum\:_{m=1}^{5}{(\delta }_{2m}* {E}_{m}+ {\delta}_{2m}^{*}* {E}_{m}*Z)]\end{aligned}$$

and assuming independent censoring (i.e., lost-to-follow-up or drop-out), the hazard for censoring is given by: ${\lambda}_{s=3}\left(t\right)=exp[{\alpha}_{3}]$. For the simulation study, cause-specific hazards were assumed to be constant over time, such that the hazard functions did not depend on time. This simplifying assumption was chosen to isolate the impact of effect modification and moderator structure. The baseline log-hazards for each of the events are denoted ${\alpha}_{1}, {\alpha}_{2},$ and ${\alpha}_{3}$. The treatment effect for each of the clinical events is ${\beta}_{1}$ and ${\beta}_{2}$. The main effects and effect modification for each of the five effect moderators is ${\delta}_{sm}$ and ${\delta}_{sm}^{*}$, respectively. We first generate a cause-specific event time ${S}_{1}$ from the all-cause hazard ${\lambda}_{s=1}\left(t\right)+ {\lambda}_{s=2}\left(t\right)+ {\lambda}_{s=3}\left(t\right)$ and the type of event at ${S}_{1}$ is based on the multinomial probabilities determined by the hazards of the event type:

11 $$\mathrm{Pr}\left(non-fatal\:event\right)= \frac{{\lambda}_{s=1}\left({ S}_{1}\right)}{{\lambda}_{s=1}\left({ S}_{1}\right)+ {\lambda}_{s=2}\left({ S}_{1}\right)+ {\lambda}_{s=3}\left({ S}_{1}\right)}$$

12 $$\mathrm{Pr}\left(death\right)= \frac{{\lambda}_{s=2}\left({ S}_{1}\right)}{{\lambda}_{s=1}\left({ S}_{1}\right)+ {\lambda}_{s=2}\left({ S}_{1}\right)+ {\lambda}_{s=3}\left({ S}_{1}\right)}$$

13 $$\mathrm{Pr}\left(censoring\right)= \frac{{\lambda}_{s=3}\left({ S}_{1}\right)}{{\lambda}_{s=1}\left({ S}_{1}\right)+ {\lambda}_{s=2}\left({ S}_{1}\right)+ {\lambda}_{s=3}\left({ S}_{1}\right)}$$

The administrative censoring time is set at 4 years and applied uniformly to all participants; subsequently any cause-specific event time ${S}_{1}\ge 4$ is truncated. The simulation uses follow-up time since each participant’s baseline time and therefore assumes uniform maximum follow-up duration rather than a fixed calendar study closure date. In trials with staggered recruitment and calendar-time study closure, shorter follow-up among later-enrolled participants may influence the observed EWTR outcome, particularly when the risk of component events changes over follow-up time. In this setting, if the outcome at time ${S}_{1}$ is either death or censoring, the simulation is completed for this participant; however, if it is the non-fatal event then the simulation will continue by generating an incremental event time ${S}_{2}$ and the multinomial probabilities for the event at time ${S}_{1}+ { S}_{2}$ for either death or censoring. We adjust the all-cause hazard by excluding ${\lambda}_{s=1}\left({ S}_{1}\right)$ from the denominator. We refer the readers to Troendle et al. for an example of three clinical events [20]. 

#### Effect modification

Effect moderators ${E}_{1}, {E}_{2},$ and ${E}_{3}$ were generated under a standard normal distribution and ${E}_{4}$ and ${E}_{5}$ were generated under a binomial distribution assuming *p=0.7* and *p=0.3*, respectively. Binary variables were generated as 0/1 but effect-coded in the moderator analyses. The null and various effect modification scenarios are shown in Table 1, along with the parameter values. We assume that the main effect and effect modification is common across the two clinical event models. Treatment effects are assumed to be consistent across both clinical event models; thus, the scenarios do not reflect differential treatment effects between non-fatal and fatal endpoints. For the null scenarios, we considered no main effect for each moderator (${\delta}_{sm}=0$), or some main effect for each moderator (${\delta}_{s1}=0.3, {\delta}_{s2}=0.02, {\delta}_{s3}=-0.5, {\delta}_{s4}=-0.4, {\delta}_{s5}=0.6$). The magnitude of the effect modification is quantified by the ratio of the interaction effect to the main effect, denoted $\Phi =\frac{{\delta}_{sm}^{*}}{{\delta}_{sm}}.$ Under the alternative scenarios, *Φ* takes the value $\frac{{\delta}_{sm}^{*}}{{\delta}_{sm}}$. A ratio of 1 indicates that the interaction effect has the same magnitude as the main effect of its respective moderator. Ratios > 1 indicates a stronger interaction effect (relative to the main effect) and ratios < 1 indicates a weaker interaction effect and/or more main effect. For instance, an interaction effect can exceed the main effect when the moderator has little impact on the overall outcome but substantially changes the treatment benefit between subgroups. Across all alternative scenarios, we evaluated ratios *Φ* of 4, 2, 1, and 1/2.

**Table 1:** Effect modification parameter values for null and alternative scenarios

**# Scenario**	${{\boldsymbol{\delta}}}_{{\boldsymbol{s}}1}^{\boldsymbol{*}}$	${{\boldsymbol{\delta}}}_{{\boldsymbol{s}}2}^{\boldsymbol{*}}$	${{\boldsymbol{\delta}}}_{{\boldsymbol{s}}3}^{\boldsymbol{*}}$	${{\boldsymbol{\delta}}}_{{\boldsymbol{s}}4}^{\boldsymbol{*}}$	${{\boldsymbol{\delta}}}_{{\boldsymbol{s}}5}^{\boldsymbol{*}}$
1. No effect modification	0	0	0	0	0
2. Mix of strong, weak and no effect modification	0.80	−0.25	0	−0.80	0.35
3. Weak effect modification	0.10	−0.12	0.13	−0.15	0.15
4. One strong and four weak effect modification	−0.80	−0.15	0.10	−0.15	0.15
5. Moderate effect modification	0.25	−0.30	0.35	−0.40	0.45

The effect modification parameter values (${\delta}_{sm}^{*}$) for each of the five effect moderators are shown and correspond to the specified scenarios. Effect moderators ${E}_{1}, {E}_{2},$ and ${E}_{3}$ were generated under a standard normal distribution and ${E}_{4}$ and ${E}_{5}$ were generated under a binomial distribution. Treatment effect parameter values for the CSH models: ${\beta}_{1}={\beta}_{2}=-0.25$

In the alternative scenarios, we also used the Gail-Simon test for qualitative interactions across the simulated trials for each moderator and for the composite moderator M* [10]. Continuous moderators were stratified into by tertiles and quartiles while binary moderators were kept as is for this test. Empirical rejection rates for qualitative interactions are detailed in Table A1. Scenarios 2 and 4 consisted of at least one moderator with predominantly qualitative interactions, and when these were combined with other moderators with quantitative interactions, the composite moderator was generally qualitative. Though in scenarios with decreasing ratio magnitudes, quantitative interactions became the predominant pattern. For Scenarios 3 and 5, the individual moderators were all mostly quantitative. The composite moderator remained quantitative for Scenario 3, however for Scenario 5 with higher ratio magnitudes, the composite moderator shifted to a more qualitative interaction pattern.

#### Additional scenarios

We simulated additional scenarios to evaluate the robustness of this extended approach.

1. Increased sample size to *N* = 2000 to assess whether the performance metrics improved and whether there were discernable differences compared to the main results.
2. Correlated moderators were generated from multivariate normal distributions with prespecified covariance structure (low-moderate pairwise correlations; see matrices in Table A2).
3. Varied the number of moderators by doubling the number of moderators (five weak and five moderate). We also explored subsets of moderators to rebuild the composite moderator to mimic real-world constraints, availability, and/or knowledge of selecting moderators from a larger set of moderators.
4. Inclusion of an endpoint with null treatment effect (${\beta}_{2}=0, {\delta}_{2m}^{*}=0$) and subsequently no effect modification for the fatal clinical event model. We hypothesized a setting where one includes a composite outcome that includes a null treatment effect on one of the endpoints while the other model has a treatment effect and effect modification on the other clinical event model (consistent with the CSH model parameters in Table 1). This contrasts with the primary setting where treatment effects are assumed to be consistent across endpoints.
5. A single clinical endpoint (one CSH model), with a treatment effect and effect modification following the patterns described in Table 1. We hypothesized a setting where the clinical interest is on the fatal event endpoint.

#### Parameter settings

The following parameter values were selected for the CSH models: $\psi =0.5, {\alpha}_{1}=-0.5{, \alpha }_{2}=-1.6, {\gamma}_{1}={\gamma}_{2}=1,$ and ${\alpha}_{3}=-4$. With *ψ =0.5,* we hypothesized that the frailty *U* influences the event’s CSHs and *γ* indicates the shared log-hazard frailty between the event models for an individual. The parameter values ${\alpha}_{1}$ and ${\alpha}_{2}$ are the baseline log-hazard for the respective clinical events. There is assumed to be a treatment effect for both clinical events, ${\beta}_{1}={\beta}_{2}=-0.25$, however in additional analyses, we evaluated simulations assuming treatment effect for one clinical event but not the other ${\beta}_{1}=-0.25, {\beta}_{2}=0$. In addition, we assessed the performance of using only one clinical event with an assumed treatment effect.

### Personalized treatment recommendation

**Fig. 1:**
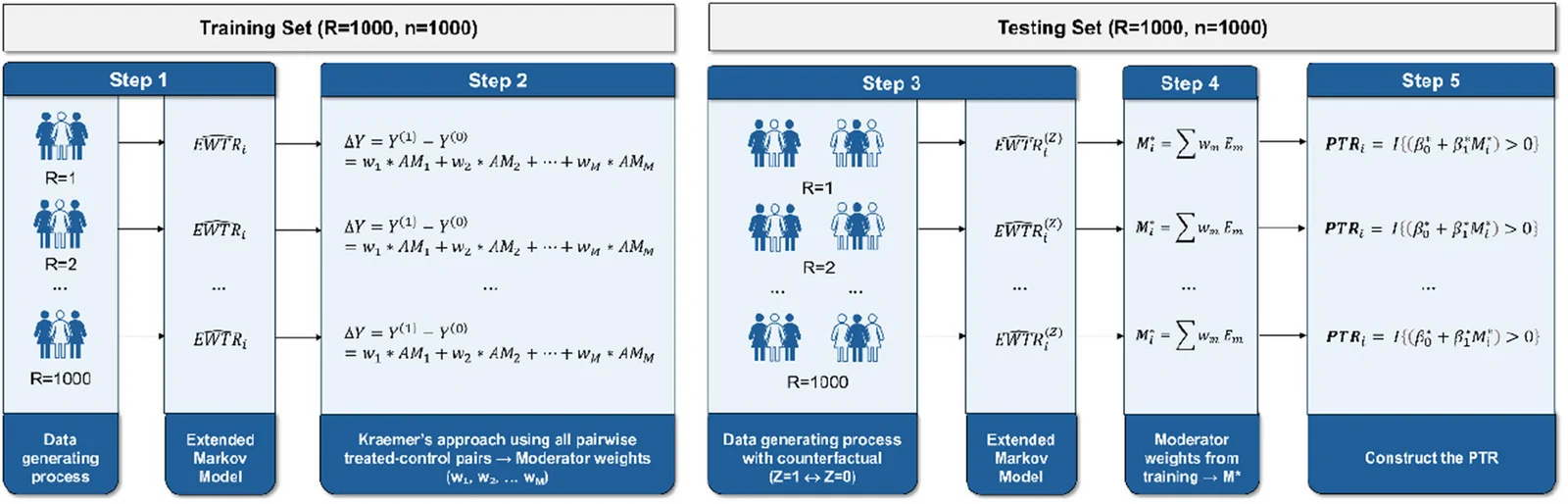
Workflow diagram for constructing the PTR from simulations. EWTR = expected win time against reference; PTR = personalized treatment recommendation. Simulations generated for R = 1000 trials of size *N* = 1000. Coefficients (${{\boldsymbol{\beta}}}_{0}^{\mathbf{*}},{{\boldsymbol{\beta}}}_{1}^{\mathbf{*}}$) for Step 5 is from a regression model with treatment (${\boldsymbol{Z}}$) and treatment-moderator interaction terms (Eq. 14)

First, training datasets (R = 1000 trials of size *N* = 1000) were simulated following the above CSH models and effect modification parameter values for each scenario (Fig. 1). For each trial in the training dataset, we transformed the two clinical time-to-event outcomes to an ordered composite endpoint by estimating the clinical state distribution via extended Markov model to derive an EWTR for every participant *i*. In Kraemer’s moderator analyses the treatment variable was re-parameterized using effect coding, $Z \in \left\{-\frac{1}{2},\frac{1}{2}\right\},$and we fit a linear model using all pairwise outcome differences and average moderator values across pairs of treated and control participants [13]. After estimating the weights for each effect moderator included in the model, we simulated testing datasets following the same specifications as the training sets but with a different replication seed, allowing evaluation of the PTR on independent data to reduce overfitting. In simulating the testing datasets, participants would be randomly assigned to either treatment or control arm. In the same replicate, while keeping all else the same, we switched the treatment assignment to generate a counterfactual outcome. This allows us to define an individual’s optimal strategy (i.e., treatment yielding the larger EWTR) and to evaluate PTR performance relative to this benchmark, which is not observable in practice. Then, we used the same process to derive EWTR for each participant, obtaining the expected outcome if all participants were assigned to the control strategy and the expected outcome if all participants were assigned to the treatment strategy. Using the weights previously estimated in the training data, we compute the composite moderator M* for each participant in the testing set. We fit a linear model on the subject-level with the EWTR as the outcome, treatment (*Z*), composite moderator (${M}^{*}$), and the interaction between treatment and the composite moderator:

14 $${\widehat{EWTR}}_{i}={\alpha}_{0}+{{\alpha}_{1}M}^{*}+Z\left({\beta}_{0}^{*}+{\beta}_{1}^{*}{M}^{*}\right).$$

We use these parameters to compute the PTR:

15 $$PTR= I\left\{\left({\beta}_{0}^{*}+{\beta}_{1}^{*}{M}^{*}\right)>0\right\}$$

where *I()* is the indicator function for recommending the treatment strategy over the control strategy for the participant when the bracket expression is true [4].

### Performance measures

For each scenario, we assessed the following four performance measures. We report on the empirical rejection rate, the proportion of simulated trials in which the treatment *×* composite moderator M* interaction coefficient was non-zero at *α =0.05* with EWTR as the outcome in the testing dataset. The rejection rate is the type I error for null effect modification scenarios and in alternative scenarios, it is the power to detect effect modification. The misclassification rate is computed at the subject level by comparing the PTR to each subject’s optimal strategy, defined as the strategy with the larger EWTR from the EWTRs simulated under both control and treatment arms. In other words, it is the proportion of the simulated testing set patients in which the PTR conflicts with the patient’s known optimal strategy. We summarized the expected outcomes by averaging EWTR across the test set under the three strategies: all received treatment, all received control, and PTR. For the expected PTR, we averaged the EWTR from the arm recommended for each subject. Lastly, we considered the gain from personalization, or expected change in the outcome under a PTR compared to a strategy where all participants received treatment [4]:

16 $${\Theta}_{Treatment}= \mu \left(PTR\right)-\mu \left(Treatment\right)$$

and analogously compared to where all participants received control:

17 $${\Theta}_{Control}= \mu \left(PTR\right)-\mu \left(Control\right).$$

Annotated R code for one Scenario 5 simulation setting is provided in the Appendix to improve reproducibility, including the data-generating mechanism, EWTR calculation, composite moderator construction, generation of independent testing datasets, and testing-set evaluation used to reduce overfitting in the PTR assessment.

## Simulation results

### Rejection rate

For the null scenarios, the type I error rates for each moderator and composite moderator were close to the nominal level *α =0.05.* Despite a few individual moderators having slightly elevated type I errors, the type I error for the composite moderator M* remained stable (See details in Table A3).

**Fig. 2:**
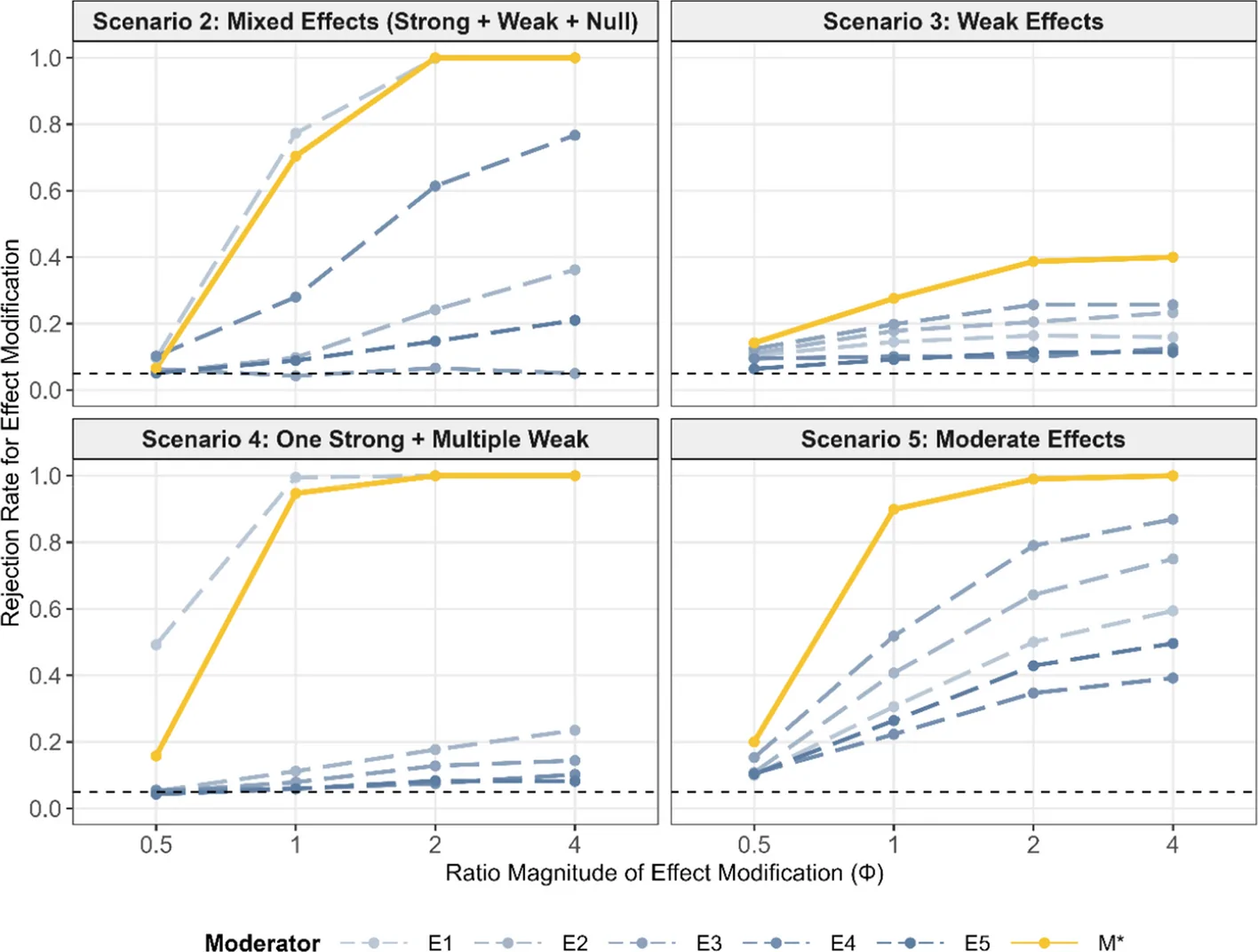
Empirical rejection rate for effect modification across varying ratio magnitudes for alternative scenarios. Black dotted line marks the nominal level ${\boldsymbol{\alpha}}=0.05$. The effect modification rejection rate is the test of the interaction coefficient for each effect moderator (${{\boldsymbol{E}}}_{1}\dots {{\boldsymbol{E}}}_{5},$) and the composite moderator M* (all separately) on the EWTR outcome across 1000 simulated trials (*N* = 1000). Lines are connected across increasing treatment-moderator interaction-to-main effect ratio magnitudes (${\boldsymbol{\Phi}}$) (i.e., stronger effect modification relative to the main effects)

In the alternative scenarios, Fig. 2 illustrates the increasing power with higher ratio magnitude (stronger effect modification) for each moderator and the composite moderator M*. When the effect modification was substantial, the composite moderator M* consistently yielded similar power to the strongest moderator, or the highest power relative to the individual effect moderators. In Scenarios 2 and 4, which included strong qualitative moderators, the power (≥ 0.70) for both the strongest individual and composite moderator M* rapidly increased at a ratio magnitude *Φ* ≥ 1. In Scenario 5, all individual moderators gradually increased in power with higher ratio magnitude *Φ*. Notably, the composite moderator M* had a steep increase in power at a ratio magnitude *Φ* ≥ 1 and a shift from more quantitative to qualitative interaction patterns. For Scenario 3, which was largely absent of qualitative interactions, the power for all moderators increased modestly with ratio magnitudes and the composite moderator M* generally outperformed the individual moderators.

### Misclassification rate under personalized treatment recommendation strategy

**Fig. 3:**
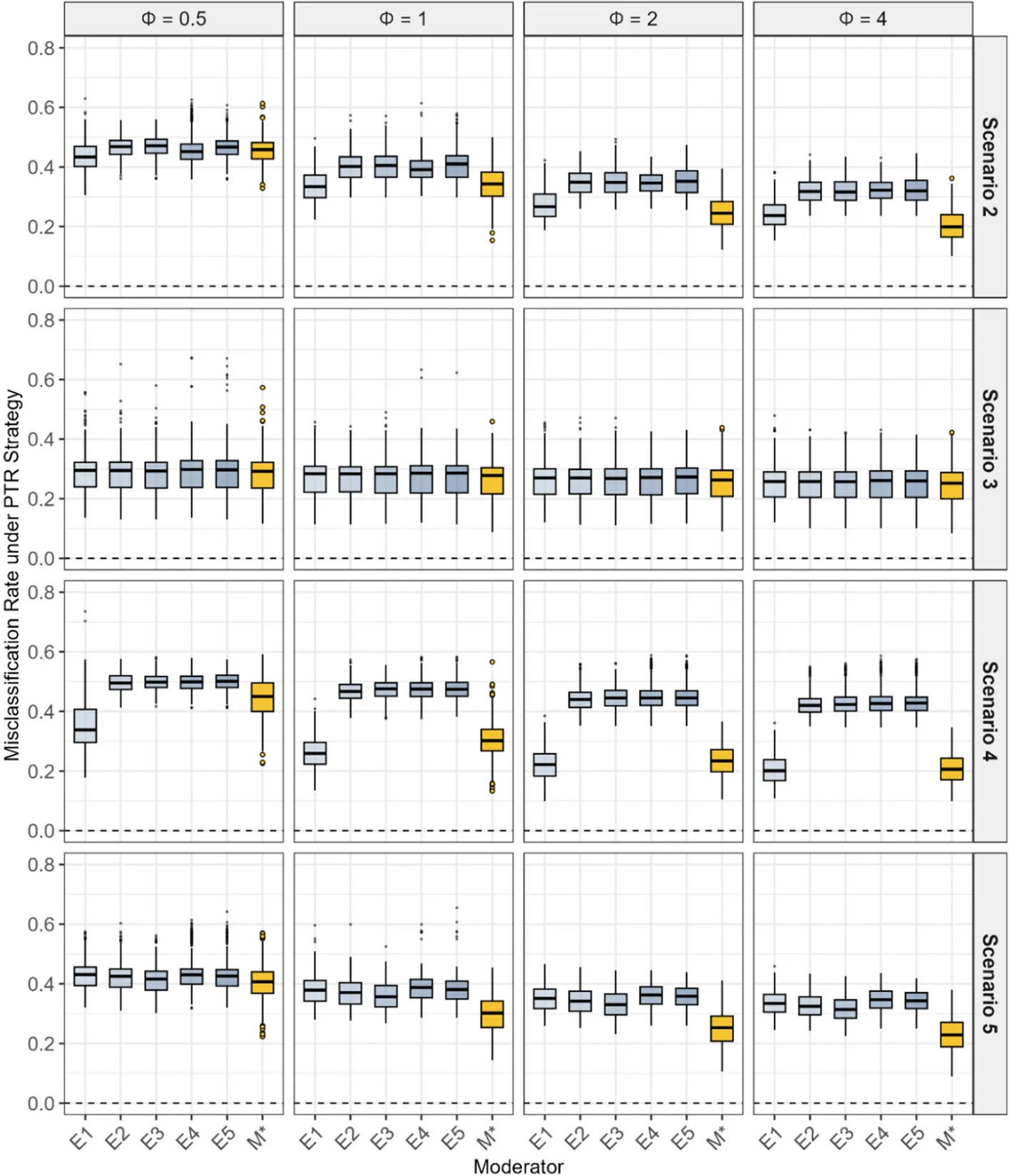
Misclassification rate across ratio magnitudes of effect modification for alternative scenarios. Boxplots show the distribution of misclassification rate for 1000 simulated trials (*N* = 1000). Misclassification is computed by comparing PTR to each subject’s ‘oracle’ optimal strategy, defined as the strategy with the larger EWTR (EWTRs simulated under both treatment arms). Black dotted line marks the misclassification at zero. Columns left to right increase in the treatment-moderator interaction-to-main effect ratio. Each row represents a scenario. Blue-gray boxplots are individual moderators, and yellow boxplots are the composite moderators M*

The distribution of misclassification rate by moderators across ratio magnitudes for the alternative scenarios is shown in Fig. 3. As the ratio magnitude *Φ* increases, or effect modification strengthens, we generally observe lower misclassification. We observe this pattern particularly for Scenarios 2, 4, and 5. In Scenarios 2 and 5, the composite moderator M* has similar, if not a lower misclassification rate relative to individual moderators. In Scenario 4, the composite moderator M* was competitive with the strongest moderator, particularly under the presence of strong effect modification. In Scenario 3, the individual moderators and the composite moderator M* had similar misclassification rates.

### Expected outcomes and expected change in the outcome under personalized treatment recommendation strategy

**Fig. 4:**
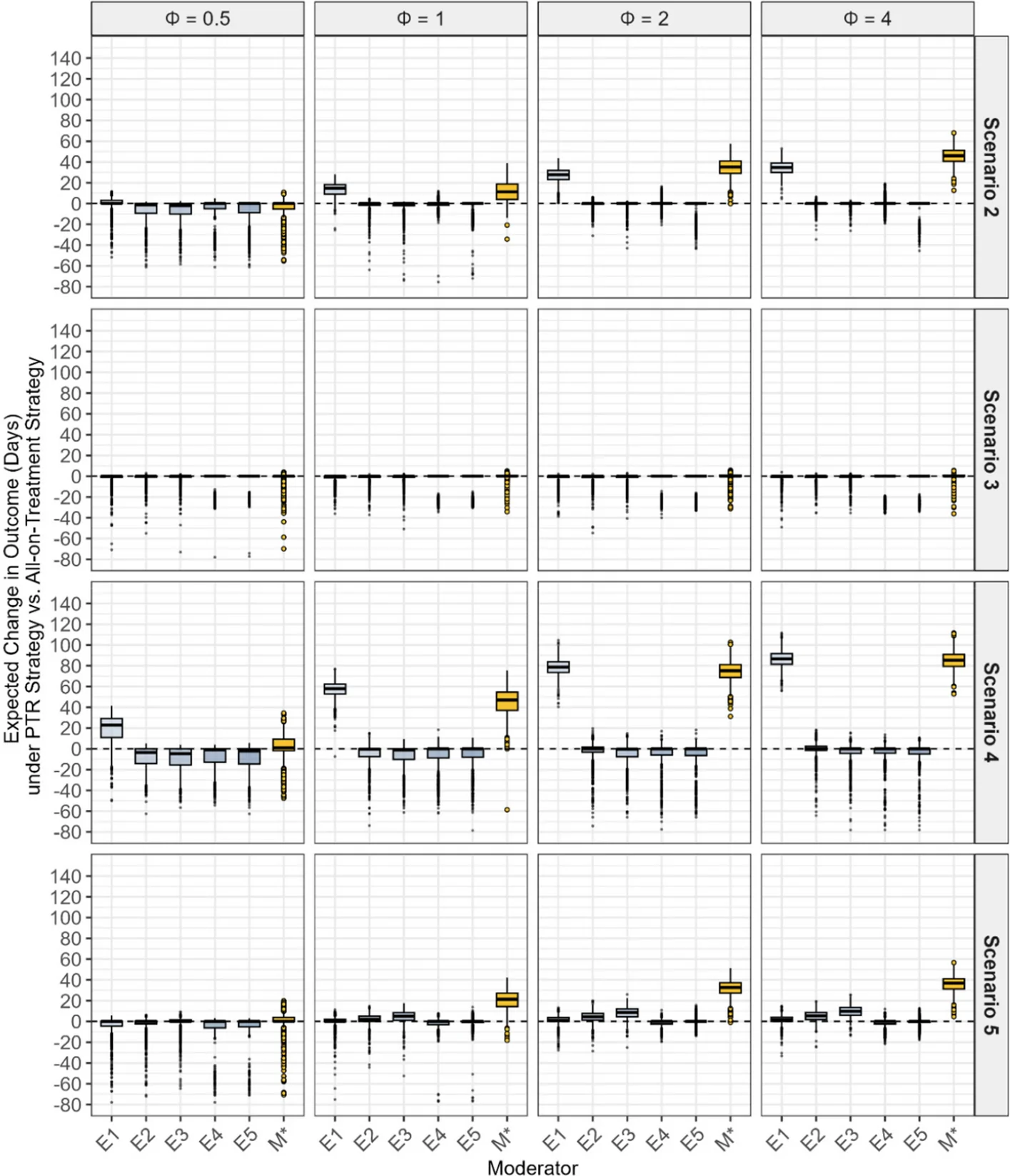
Expected change in outcome under a PTR strategy compared to all-on-treatment strategy across ratio magnitudes of effect modification for alternative scenarios. Boxplots show the distribution of the gains from personalization or expected change in outcome under PTR versus all-on-treatment strategy for 1000 simulated trials (*N* = 1000). Black dotted line marks the change at zero. Columns left to right increase in the treatment-moderator interaction-to-main effect ratio. Each row represents a scenario. Blue-gray boxplots are individual moderators, and yellow boxplots are the composite moderators M*

We compared the expected EWTR under the three treatment strategies: all-treatment, all-control, and PTR. We illustrate the gain from personalization or expected change in the outcome under a PTR compared to a strategy where all participants received treatment (${\Theta}_{Z=1}$), for each individual moderator and the composite moderator M* in Fig. 4. In Scenarios 2 and 5 the composite moderator M* matched, if not exceeded the individual moderators in the gains under the PTR strategy relative to having all participants on treatment strategy. In Scenario 4, the composite moderator M* was competitive with the strongest moderator in the gains under PTR strategy with higher ratio magnitude. Across the three scenarios, gains under the PTR strategy relative to non-personalized strategies – for the composite moderator M* or the strongest individual moderator – increased with higher ratio magnitude and followed the shift toward a more qualitative interaction pattern. For Scenario 3, the gain from personalization was minimal relative to assigning all participants to treatment strategy across the individual moderators and the composite moderator M*.

### Sensitivity and additional scenarios

Simulation results for the larger trial size of *N* = 2000 can be found in Table A4, Figures A1-2; results are described in comparison to the primary setting (*N* = 1000). For the rejection rates, the type I error was slightly elevated for the null scenarios and power to detect effect modification increased across the alternative scenarios. In general, the misclassification rate was slightly lower across both the individual and composite moderators. For scenarios and moderators that previously had gains from personalization under size *N* = 1000, this magnitude increased. For scenarios and moderators that had minimal gain in personalization under a smaller sample size remained consistent. A previous study demonstrated the Kraemer composite moderator framework using a real randomized trial with a moderate sample size (*N* = 291) [14].

In early applications of the Kramer approach, moderators were selected based on the assumption of minimal correlation between them [14]. Our simulations evaluating mild to moderate correlation between moderators found that the null scenario was maintained at the nominal level and power for the alternative scenarios were generally similar or attenuated slightly. It was notable that the misclassification rate was higher and the gains under personalization were reduced, and more variability in the latter, than the setting without correlation between the individual moderators (Table A5, Figures A3-4).

In simulations with ten moderators, the addition of five weak moderators to the presence of five stronger effect moderators did not improve the power, misclassification rate, or provide substantial gains for the composite moderator M* under the PTR strategy (Table A6, Figures A5-6). When we employed an ‘oracle’ moderator selection, defined as restricting to only the strongest moderator(s) based on the known data-generating mechanism for inclusion in the composite moderator, the performance was similar or slightly improved compared with the ordinary composite moderator (results not shown).

Moreover, we performed simulations assessing changes to the CSH models to evaluate its robustness under two settings (i) included a CSH model with a null treatment effect and subsequently no effect modification on one of the clinical events and (ii) used a single clinical endpoint but consistent treatment and effect modification parameters. In setting (i) the inclusion of a CSH model with no effect modification on one of the clinical events while the other clinical event parameters remain unchanged, worsened overall performance and substantially reduced gains for single or composite moderators under PTR strategy (Table A7, Figures A7-8). In setting (ii) the use of a single endpoint (assuming same effect modification parameters) could be advantageous, with substantial increases in power to detect effect modification for single and composite moderators with stronger ratio magnitudes, however the tradeoff is a higher misclassification rate and attenuated gains under the PTR strategy (Table A8, Figures A9-10).

## Application

The AFFIRM (Atrial Fibrillation Follow-Up Investigation of Rhythm Management) study was a randomized trial comparing two treatment approaches for atrial fibrillation (AF) [27]. It enrolled 4060 AF patients with a high risk of stroke or death and patients were randomly assigned to rate-control strategy (*N* = 2027) or rhythm-control strategy (*N* = 2033). Patients were on average 70 years old (SD 9) and male (60.7%). The primary endpoint was overall mortality, and the mean follow-up time was 3.5 years with a maximum of 6 years. There were more deaths in the rhythm-control group relative to rate-control group, however the difference was not statistically significant [356 events vs 310 events, HR 1.15 (95% CI 0.99–1.34), *p* = 0.08] after accounting for interim monitoring [27]. The authors in the study noted that patients in the rhythm-control group were more likely to be hospitalized and have adverse drug effects relative to the rate-control group [27]. In a pre-specified subgroup analysis, rhythm-control strategy was related to a higher risk of death than rate-control among older adults (≥ 65 years), those without congestive heart failure (CHF), and those with coronary artery disease (CAD). The pre-specified baseline covariates used in the subgroup analyses (e.g., age, CHF, CAD, left ventricular ejection fraction (LVEF), and hypertension) were adjusted for in a Cox proportional hazards model evaluating the risk of death comparing the two treatment strategies, which remained non-significant.

**Fig. 5:**
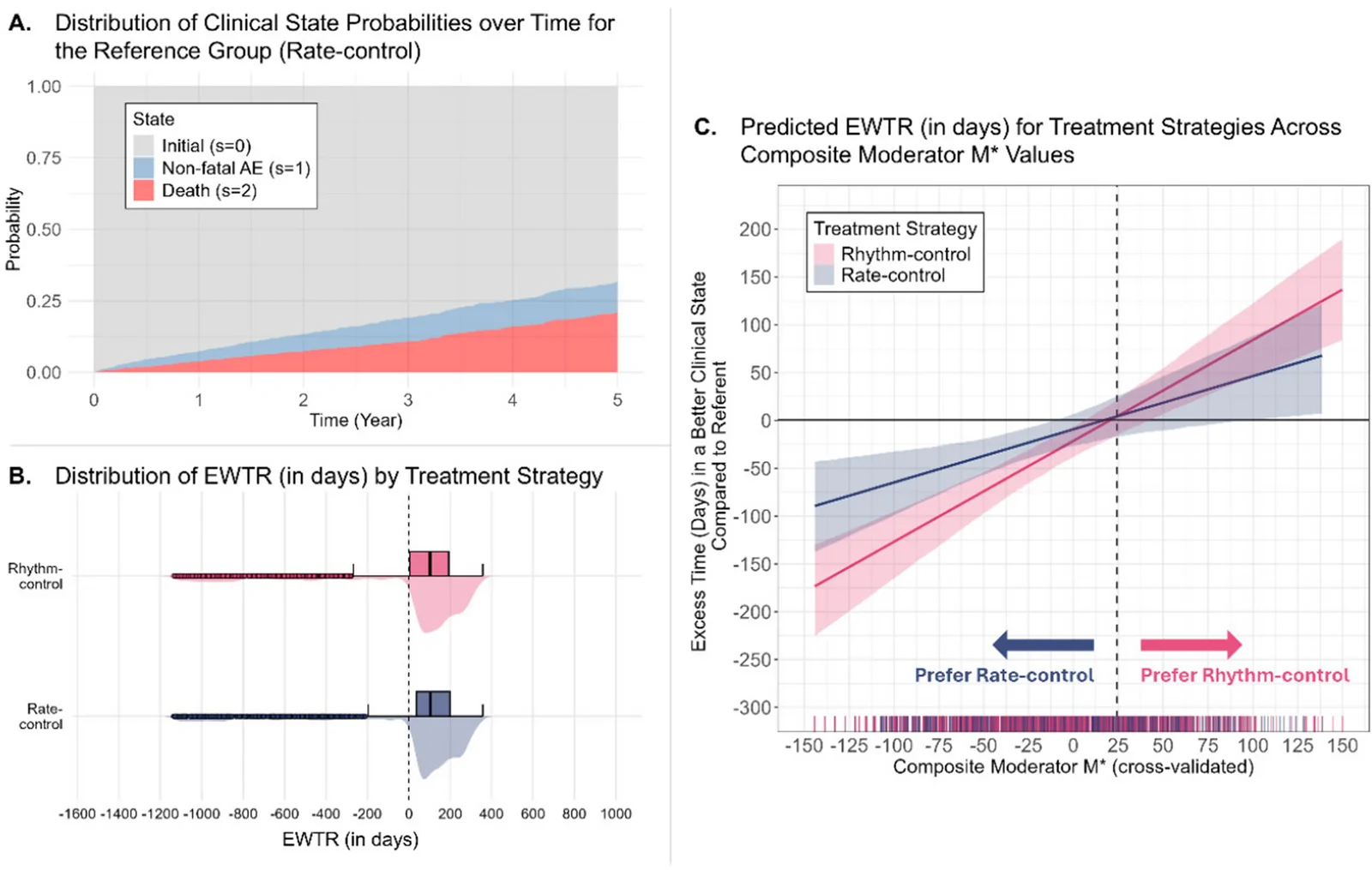
Application of composite moderator framework to the AFFIRM Trial. **A** Distribution of clinical state probabilities over time using the reference group (rate-control). Probability estimates are based on the extended Markov model. To align with publication results, data were truncated at five years. **B** Distribution of expected win time against reference (EWTR, in days) by treatment strategy. Dotted vertical line marks the EWTR at zero, indicating no difference in expected time spent in better clinical states relative to the reference. Horizontal boxplots indicate the median and interquartile range for each treatment strategy, with density shading illustrating the distribution of EWTR (in days). **C** Regression estimates were used to plot predicted EWTR for each treatment strategy across observed values of composite moderator M*, with shaded bands indicating 95% bootstrapped confidence intervals. Regression lines for rate- and rhythm-control cross at the vertical dashed line (${{M}}_{{intersection}}^{*}=24.2$), representing the decision boundary for treatment recommendation. Participants with M* values to the left are recommended rate-control, whereas those to the right are recommended rhythm-control. Rug plot along the x-axis shows the distribution of observed M* values

We re-analyzed the trial data incorporating the first adverse event (AE) event (e.g., arrhythmia, ischemic stroke, major bleeding, etc.) as a clinical state, in addition to death. Figure 5 illustrates the clinical state probability distribution over the common follow-up for the rate-control strategy (reference) and the distribution of the EWTR between the two treatment strategies. Most participants remained in the initial state, and we observe a higher probability of death relative to non-fatal AE over the duration of follow-up. In a linear model for EWTR comparing treatment strategies, rate-control had slightly more excess days in a better clinical state relative to rhythm-control (${\beta}_{EWTR}=41\ days, p=0.07)$. We selected the same pre-specified baseline covariates evaluated in the original trial —age, CHF, CAD, and hypertension—for inclusion in the composite moderator. LVEF was considered in the original trial but was not available for this analysis.

We performed a 5-fold cross validation analysis; for each training set, we estimated the weights for the four effect moderators using Kraemer’s approach and using the testing set, created a composite moderator M*. Thus, across all folds, we obtain a M* value for each participant. After fitting the following linear model:

18 $${\widehat{EWTR}}_{i}=\alpha + {\beta}_{0}*Z+ {\beta}_{1}*Z*{M}_{i}^{*}$$

regression estimates were used to plot the predicted EWTR values for rate-control and rhythm-control strategy across the observed values of M* (Fig. 5). The predicted regression lines for the treatment strategies cross at ${M}_{intersection}^{*}=24.2$ and this is within the observed range. The intersection represents the split in the two subgroups, if subjects have M* ≥ 24.2 then under PTR they would be recommended the rhythm-control strategy and if subjects have M* < 24.2 they would be recommended the rate-control strategy. In cross-validation analyses, the treatment *×* M* interaction was significant (*p* < 0.05). Using the Gail–Simon test, we found insufficient evidence of qualitative interaction on EWTR (*p* = 0.06 for 4 strata; *p* = 0.10 for 3 strata). The results instead suggested a quantitative interaction, with consistent treatment effect direction but varying magnitude across levels of M*.

We used descriptive statistics to characterize the two subgroups drawn from the four individual moderators to derive M* (Table A9). The four moderators were weighed similarly, with CHF receiving the highest weight. Younger participants and those with CHF were generally recommended the rhythm-control strategy (M* ≥ 24.2), while participants with CAD and hypertension were generally recommended the rate-control strategy (M* < 24.2). To characterize participant profiles and highlight uncertainty in recommendations for M* values near the intersection, we explored a Gaussian-mixture model to cluster M* (Table A10).

**Fig. 6:**
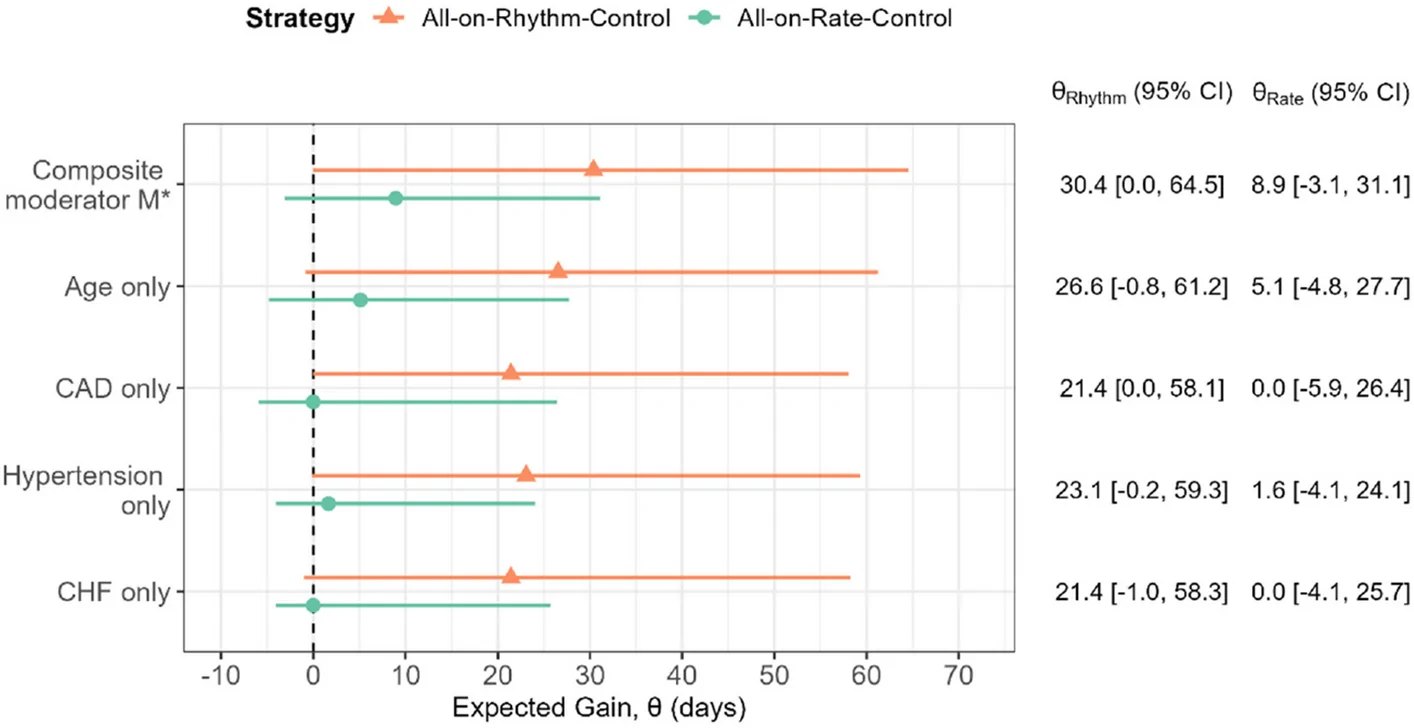
Expected gain under PTR strategy compared with non-personalized strategies. Estimated expected gain (θ) under a personalized treatment recommendation (PTR) is compared with assigning all participants to rhythm- or rate-control. The 95% percentile confidence intervals are computed from B = 1000 bootstrapped samples [28]. Dashed vertical line at zero denotes no gain relative to the non-personalized strategy. Gains are defined as: ${{\Theta}}_{{Rhythm}}={\mu}\left({PTR}\right)-{\mu}\left({Rhythm}\right)$, the expected change in EWTR under PTR compared with assigning all participants to rhythm-control (see Eqs. 6, 7). ${{\Theta}}_{{Rate}}={\mu}\left({PTR}\right)-{\mu}\left({Rate}\right)$, the expected change in EWTR under PTR compared with assigning all participants to rate-control (see Eqs. 6, 8). Positive values indicate, on average, more days in better clinical states under PTR

The expected outcome recommending all participants on rhythm-control was worse in EWTR relative to rate-control (−40.7 [95% bootstrap CI −70.3 to −16.2] vs −19.2 [95% bootstrap CI −46.9 to 6.0] days). The gains in personalization for the single and composite moderators were noticeably higher against the all-on-rhythm-control strategy but were minimal relative to the all-on-rate-control strategy (Fig. 6). Compared to a single moderator, using the composite moderator performed the best under a PTR strategy and had the highest gains in personalization relative to all-on-rhythm or all-on-rate control strategies.

## Discussion

We extended Kraemer’s composite moderator framework to time-to-event outcomes by using EWTR, an estimate used to capture the clinical experience of a participant through time as they progress from less to more severe events. Under the stratified medicine paradigm, we used the composite moderator to construct a PTR strategy to identify subgroups for whom a given treatment strategy is expected to yield greater benefit. In simulations the type I error was maintained; the power, misclassification rate, and expected outcome gains under PTR improved as effect modification strengthened. In the presence of strong effect modification, the composite moderator often matched or outperformed the best single moderator. Its performance was attenuated by weak interactions, correlated moderators, and the inclusion of an endpoint unaffected by treatment moderation.

In randomized trials, heterogeneity of treatment effects manifests as quantitative or qualitative interaction, with the latter not commonly observed but presenting serious concerns for the interpretation of study results [10–12]. As such, the detection of qualitative interactions in a trial with significant findings can provide a rationale to limit future treatment use to specific subgroups(s) that benefit. Quantitative interactions, however, are anticipated but generally carry less clinical importance. In our simulations we calibrated settings under both conditions; qualitative patterns often paralleled higher ratio of interaction-to-main effect magnitudes. Our findings show the highest power, lowest misclassification, and highest gains in the expected outcome under personalization, using either a single or composite moderator, are in trials with strong qualitative patterns or high ratio magnitudes. With quantitative interactions or low ratio magnitudes, the performance metrics under personalization were often modest. While we demonstrate the strength of effect modification required to observe gains from a personalization strategy, it is acknowledged that many real-world trials may not present magnitudes of effect modification to observe these benefits. Although our simulations were based on sample sizes of 1,000 and 2,000, which are moderately large for a late-phase cardiovascular trial, smaller trials may have less power to detect effect modification and may therefore struggle to demonstrate meaningful gains from personalization. Clinical trials are not typically powered to examine subgroup or interaction effects, and as other authors have noted, it is often a challenge to expect PTR gains in small sample sizes [1, 29, 30].

The original composite moderator framework proposed by Kraemer was developed for continuous outcomes, where weights maximize the association between pairwise outcome differences and average moderator values. In extending this framework to time-to-event outcomes, we retained this principle by relating EWTR to the average moderator value within pairs, maintaining interpretability. Alternative summaries of moderator values (e.g., nonlinear transformations) may be considered and could be advantageous in certain settings; evaluating such approaches is an important direction for future research.

Our simulations revealed two roles for the composite moderator M* proposed by Kraemer [13]. In settings with weak moderators or quantitative interaction patterns (such as Scenario 3), where the true effect modification signal is distributed across several moderators, we observe an increased power to detect effect modification for the composite relative to the single moderators. However, as a decision rule under a PTR strategy, it only minimally improved misclassification rates or expected change in EWTR. Conversely, with moderate-to-strong moderators or qualitative interaction patterns, the composite moderator matched or outperformed single moderators in detecting effect modification. As a decision rule, this extended to better performance under a PTR strategy relative to non-personalized strategies. These results highlight the composite moderator’s role in detecting effect modification but depending upon the interaction pattern, may not necessarily convey improved gains in personalization. The evaluation of the PTR in this study is model-based. Alternative approaches that estimate the value of a treatment rule, defined as the expected outcome if all individuals were treated according to the rule in the optimal treatment regime framework, as well as trial emulation methods, may provide a more direct assessment of clinical benefit relative to non-personalized strategies. Extending the proposed framework to such approaches represents an important area for future research.

Our sensitivity analyses revealed several important issues to consider before implementing such an approach. First, correlation among moderators can attenuate both moderator detection and PTR performance. While the individual moderator choice should be biologically or scientifically justified, thoughtful pre-specification and screening should be prioritized to minimize redundancy between moderators when used to construct the composite moderator. Second, the addition of weak moderators in the presence of moderate-to-strong moderators does not necessarily amplify the detection nor PTR performance and instead may dilute the signal. Our findings suggest that selecting a parsimonious set of moderate-to-strong, low-collinearity moderators is preferable to including many weak moderators, consistent with prior cautions against incorporating too many irrelevant predictors [1, 11, 22, 23, 31–33]. Future work could explore regularized or shrinkage-based approaches to handle correlated moderators or settings with many candidate moderators. The composite moderator framework and the resulting PTR rely on linear and additive modeling assumptions. While these provide practical and interpretable summaries of effect modification and treatment decision rules, the assumptions may be difficult to verify and deviations may affect performance. In practice, both the composite moderator and PTR should be viewed as approximations to potentially more complex relationships. Sensitivity analyses (e.g. alternative functional forms) and external validation are important to assess robustness. Moreover, the simulation study assumed constant cause-specific hazards. While this provides a controlled setting, time-varying hazards may influence the performance of both the composite moderator and PTR and warrant further investigation.

In addition, this work demonstrates the utility of the EWTR as a potential outcome for time-to-event clinical trials. This allows use of an ordered set of clinical events within a single endpoint which can be especially helpful in longer term clinical trials where multiple events can occur to participants. In this paper we have considered the reference, or control, group to provide the reference distribution. However, this approach could also be considered for historical control data to ensure that evolving treatment regimens are compared to a consistent reference.

Furthermore, another consideration is the endpoint construction. Our results showed that conclusions on the detection and performance can depend on the inclusion of each component event, component-level treatment effects, and the choice of the clinical states. Sensitivity analyses should supplement applications of this approach to ensure robustness and clinical plausibility, as prior studies have shown that PTR may be sensitive to the underlying data-generating distribution [1, 4].

The application of this approach to the AFFIRM trial showed that the composite moderator constructed from age, CAD, hypertension and CHF yielded personalization gains over non-personalized strategies, however the percentile bootstrapped CIs are wide, indicating high uncertainty in its practical impact. While we were able to profile participants in the respective subgroups using the composite moderator, it may be fruitful to characterize participants near the decision boundary (intersection), as these individuals typically drove misclassification and may benefit from additional clinical decision-making. Nonetheless, previous work exploring treatment moderators has demonstrated value of such analyses and could be extended to evaluate the potential benefits of identifying moderators, such as impact on clinical decision-making or role in designing more cost-effective future trials [3, 34–36]. These exploratory analyses may serve as guides for improving trial design and resource allocation [2, 3].

The theoretical foundations for combining win-time–based estimands such as EWTR with personalized treatment decision rules are still evolving. In this work, EWTR is used as a summary outcome to capture time spent in better clinical states, and the resulting PTR should be interpreted as an empirical decision rule. Further work is needed to formally evaluate the theoretical properties of this framework. The identification of qualitative interactions also raises important ethical considerations, particularly in trials designed under the assumption of an overall treatment benefit in a broad population. Such findings should be interpreted cautiously and, if validated, may inform more targeted treatment strategies but potentially restrict the population of patients eligible in future studies. If it is possible to validly identify patient subgroups unlikely to benefit from a given treatment, this could help avoid exposing these patients to less effective therapies, representing a potential ethical benefit. However, prospective validation is essential before such subgroup-specific recommendations are considered for clinical or trial design implementation. The proposed composite moderator framework and resulting PTR should be interpreted as exploratory and hypothesis-generating. In this context, the approach is intended to identify potential heterogeneity of treatment effect and inform future trial design, subgroup identification, while still requiring external validation, rather than confirming subgroup-specific treatment superiority. In a personalized treatment paradigm, external validation is essential [1, 15, 30–33]; independent trials are needed to confirm that observed effect modification translates into overall benefit under a PTR strategy prior to clinical implementation.

## Conclusion

We extended Kraemer’s composite-moderator framework for time-to-event endpoints via EWTR and showed that when the effect modification is moderate-to-strong, the composite moderator M* often matched, if not outperformed the best single moderator. However, its performance was attenuated with weak interactions, correlated moderators, or the inclusion of a null endpoint. Collectively, these findings support the composite moderator M* as a pragmatic tool for detecting effect modification while clarifying under what conditions a PTR strategy is likely to be beneficial.

## Supplementary Information

Supplementary Material 1.

Supplementary Material 2.

Supplementary Material 3.

Supplementary Material 4.

## Data Availability

The AFFIRM clinical trial data used in this study were requested and approved through the National Institute of Health (NIH) BioLINCC program (https://biolincc.nhlbi.nih.gov/home/).
