# Supplementary material for: Leveraging win time endpoint and combining moderators for personalized treatment recommendation in time-to-event clinical trials
Source: BMC Med Res Methodol. 2026 Aug 15;26:243. doi: 10.1186/s12874-026-02960-8 (PMC13753155; doi:10.1186/s12874-026-02960-8)
Supplement: Supplementary file 1 [file 12874_2026_2960_MOESM1_ESM.docx]

**APPENDIX**

LEVERAGING WIN TIME ENDPOINT AND COMBINING MODERATORS FOR PERSONALIZED TREATMENT RECOMMENDATION IN TIME-TO-EVENT CLINICAL TRIALS

Figure A1. Misclassification Rate in Simulations Across Ratio Magnitudes of Effect Modification for Alternative Scenarios with a Larger Sample Size (N=2000)

Boxplots show the distribution of misclassification rate for 1000 simulated trials (N=2000). Misclassification is computed by comparing PTR to each subject’s ‘oracle’ optimal strategy, defined as the strategy with the larger EWTR (EWTRs simulated under both treatment arms). Black dotted line marks the misclassification at zero. Columns left to right increase in the treatment-moderator interaction-to-main effect ratio. Each row represents a scenario. Blue-gray boxplots are individual moderators, and yellow boxplots are the composite moderators M*.

Figure A2. Expected Change in Outcome Under PTR Strategy Compared to All-on-Treatment Strategy, Across Ratio Magnitude of Effect Modification for Alternative Scenarios with a Larger Sample Size (N=2000)

Boxplots show the distribution of the gains from personalization or expected change in outcome under PTR versus all-on-treatment strategy for 1000 simulated trials (N=2000). Black dotted line marks the change at zero. Columns left to right increase in the treatment-moderator interaction-to-main effect ratio. Each row represents a scenario. Blue-gray boxplots are individual moderators, and yellow boxplots are the composite moderators M*.

Figure A3. Misclassification Rate in Simulations Across Ratio Magnitudes of Effect Modification for Alternative Scenarios with Correlated Moderators

Boxplots show the distribution of misclassification rate for 1000 simulated trials (N=1000). Misclassification is computed by comparing PTR to each subject’s ‘oracle’ optimal strategy, defined as the strategy with the larger EWTR (EWTRs simulated under both treatment arms). Black dotted line marks the misclassification at zero. Columns left to right increase in the treatment-moderator interaction-to-main effect ratio. Each row represents a scenario. Blue-gray boxplots are individual moderators, and yellow boxplots are the composite moderators M*.

Figure A4. Expected Change in Outcome Under PTR Strategy Compared to All-on-Treatment Strategy, Across Ratio Magnitude of Effect Modification for Alternative Scenarios with Correlated Moderators

Boxplots show the distribution of the gains from personalization or expected change in outcome under PTR versus all-on-treatment strategy for 1000 simulated trials (N=1000). Black dotted line marks the change at zero. Columns left to right increase in the treatment-moderator interaction-to-main effect ratio. Each row represents a scenario. Blue-gray boxplots are individual moderators, and yellow boxplots are the composite moderators M*.

Figure A5. Misclassification Rate in Simulations Across Ratio Magnitudes of Effect Modification for Scenario with Additional Moderators (m=10)

Boxplots show the distribution of misclassification rate for 1000 simulated trials (N=1000). Misclassification is computed by comparing PTR to each subject’s ‘oracle’ optimal strategy, defined as the strategy with the larger EWTR (EWTRs simulated under both treatment arms). Black dotted line marks the misclassification at zero. Columns left to right increase in the treatment-moderator interaction-to-main effect ratio. Each row represents a scenario. Blue-gray boxplots are individual moderators, and yellow boxplots are the composite moderators M*.

Figure A6. Expected Change in Outcome Under PTR Strategy Compared to All-on-Treatment Strategy, Across Ratio Magnitude of Effect Modification for Scenario with Additional Moderators (m=10)

Boxplots show the distribution of the gains from personalization or expected change in outcome under PTR versus all-on-treatment strategy for 1000 simulated trials (N=1000). Black dotted line marks the change at zero. Columns left to right increase in the treatment-moderator interaction-to-main effect ratio. Each row represents a scenario. Blue-gray boxplots are individual moderators, and yellow boxplots are the composite moderators M*.

Figure A7. Misclassification Rate in Simulations Across Ratio Magnitudes of Effect Modification for Alternative Scenarios with the Inclusion of an Endpoint with Null Treatment Effect

Boxplots show the distribution of misclassification rate for 1000 simulated trials (N=1000). Misclassification is computed by comparing PTR to each subject’s ‘oracle’ optimal strategy, defined as the strategy with the larger EWTR (EWTRs simulated under both treatment arms). Black dotted line marks the misclassification at zero. Columns left to right increase in the treatment-moderator interaction-to-main effect ratio. Each row represents a scenario. Blue-gray boxplots are individual moderators, and yellow boxplots are the composite moderators M*.

Figure A8. Expected Change in Outcome Under PTR Strategy Compared to All-on-Treatment Strategy, Across Ratio Magnitudes of Effect Modification for Alternative Scenarios with the Inclusion of an Endpoint with Null Treatment Effect

Boxplots show the distribution of the gains from personalization or expected change in outcome under PTR versus all-on-treatment strategy for 1000 simulated trials (N=1000). Black dotted line marks the change at zero. Columns left to right increase in the treatment-moderator interaction-to-main effect ratio. Each row represents a scenario. Blue-gray boxplots are individual moderators, and yellow boxplots are the composite moderators M*.

Figure A9. Misclassification Rate in Simulations Across Ratio Magnitudes of Effect Modification for Alternative Scenarios with a Single Clinical Endpoint (one CSH model)

Boxplots show the distribution of misclassification rate for 1000 simulated trials (N=1000). Misclassification is computed by comparing PTR to each subject’s ‘oracle’ optimal strategy, defined as the strategy with the larger EWTR (EWTRs simulated under both treatment arms). Black dotted line marks the misclassification at zero. Columns left to right increase in the treatment-moderator interaction-to-main effect ratio. Each row represents a scenario. Blue-gray boxplots are individual moderators, and yellow boxplots are the composite moderators M*.

Figure A10. Expected Change in Outcome Under PTR Strategy Compared to All-on-Treatment Strategy, Across Ratio Magnitudes of Effect Modification for Alternative Scenarios with a Single Clinical Endpoint (one CSH model)

Boxplots show the distribution of the gains from personalization or expected change in outcome under PTR versus all-on-treatment strategy for 1000 simulated trials (N=1000). Black dotted line marks the change at zero. Columns left to right increase in the treatment-moderator interaction-to-main effect ratio. Each row represents a scenario. Blue-gray boxplots are individual moderators, and yellow boxplots are the composite moderators M*

Table A1. Empirical Rejection Rate for the Gail-Simon Test for Qualitative Interaction Across Simulations for Alternative Scenarios

| **Interaction-to-main effect ratio magnitude (**$\boldsymbol{\Phi}$**)** | **Moderator** | | | | | | | | **Composite moderator M*** | |
| --- | --- | --- | --- | --- | --- | --- | --- | --- | --- | --- |
|  | **E1** | | **E2** | | **E3** | | **E4** | **E5** |  |  |
|  | Strata = 4 | Strata = 3 | Strata = 4 | Strata = 3 | Strata = 4 | Strata = 3 | Strata = 2 | Strata = 2 | Strata = 4 | Strata = 3 |
| **Scenario 2** |  |  |  |  |  |  |  |  |  |  |
| **0.5** | 0.092 | 0.081 | 0.03 | 0.034 | 0.035 | 0.031 | 0.083 | 0.036 | 0.055 | 0.049 |
| **1** | 0.284 | 0.234 | 0.015 | 0.016 | 0.016 | 0.006 | 0.107 | 0.029 | 0.26 | 0.204 |
| **2** | 0.525 | 0.40 | 0.008 | 0.005 | 0.002 | 0 | 0.133 | 0.019 | 0.702 | 0.554 |
| **4** | 0.619 | 0.469 | 0.001 | 0 | 0 | 0 | 0.128 | 0.008 | 0.824 | 0.681 |
| **Scenario 3** |  |  |  |  |  |  |  |  |  |  |
| **0.5** | 0.008 | 0.009 | 0.007 | 0.006 | 0.009 | 0.007 | 0.017 | 0.012 | 0.01 | 0.012 |
| **1** | 0.006 | 0.005 | 0.011 | 0.005 | 0.012 | 0.01 | 0.013 | 0.012 | 0.016 | 0.014 |
| **2** | 0.005 | 0.006 | 0.004 | 0.002 | 0.005 | 0.008 | 0.014 | 0.019 | 0.016 | 0.013 |
| **4** | 0.003 | 0.005 | 0.003 | 0.005 | 0.01 | 0.007 | 0.02 | 0.011 | 0.019 | 0.013 |
| **Scenario 4** |  |  |  |  |  |  |  |  |  |  |
| **0.5** | 0.675 | 0.572 | 0.04 | 0.049 | 0.05 | 0.044 | 0.048 | 0.045 | 0.151 | 0.161 |
| **1** | 0.957 | 0.934 | 0.057 | 0.065 | 0.044 | 0.054 | 0.055 | 0.05 | 0.786 | 0.775 |
| **2** | 0.995 | 0.99 | 0.07 | 0.08 | 0.05 | 0.044 | 0.059 | 0.067 | 0.99 | 0.979 |
| **4** | 0.999 | 0.996 | 0.06 | 0.066 | 0.022 | 0.026 | 0.078 | 0.043 | 0.998 | 0.996 |
| **Scenario 5** |  |  |  |  |  |  |  |  |  |  |
| **0.5** | 0.035 | 0.034 | 0.041 | 0.045 | 0.063 | 0.065 | 0.069 | 0.068 | 0.081 | 0.075 |
| **1** | 0.049 | 0.045 | 0.079 | 0.073 | 0.113 | 0.12 | 0.103 | 0.129 | 0.408 | 0.360 |
| **2** | 0.043 | 0.049 | 0.082 | 0.071 | 0.155 | 0.139 | 0.104 | 0.16 | 0.616 | 0.522 |
| **4** | 0.044 | 0.044 | 0.072 | 0.069 | 0.169 | 0.134 | 0.105 | 0.137 | 0.659 | 0.573 |

Moderators E1-E3 are continuous and E4-E5 are binary. Continuous moderators (and composite moderator M*) are stratified into subgroups of 4 and 3, then tested for qualitative treatment-moderator interaction using the Gail-Simon test (Gail, 1985) across 1000 simulated trials (N=1000). Higher empirical rejection rates suggest stronger evidence of qualitative interaction at $\alpha=0.05$.

Table A2. Correlation Matrices Among Moderators Under Additional Scenario Simulations

| **Scenarios 1, 2, 3, and 5** | **Scenario 4** |
| --- | --- |
| $\boldsymbol{\rho=}\left[ \begin{matrix} 1 & 0.05 & 0.10 & 0.15 & 0.20 \\ 0.05 & 1 & 0.15 & 0.25 & 0.30 \\ 0.10 & 0.15 & 1 & 0.05 & -0.15 \\ 0.15 & 0.25 & 0.05 & 1 & 0.20 \\ 0.20 & 0.30 & -0.15 & 0.20 & 1 \end{matrix} \right]$ | $\boldsymbol{\rho=}\left[ \begin{matrix} 1 & 0.05 & 0.10 & 0.15 & 0.60 \\ 0.05 & 1 & 0.15 & 0.25 & 0.30 \\ 0.10 & 0.15 & 1 & 0.05 & -0.15 \\ 0.15 & 0.25 & 0.05 & 1 & 0.20 \\ 0.60 & 0.30 & -0.15 & 0.20 & 1 \end{matrix} \right]$ |

In simulating correlation among moderators, we assume a multivariate normal distribution with the above correlation matrices. For binary variables (E4, E5), we used cutoffs via z standard normal value that matched the binary proportions in the main simulations. For p=0.70, z>-0.524 and p=0.30, z>0.524.

Table A3. Empirical Rejection Rate (Type I Error) for Effect Modification Under Scenario 1

| **Scenario 1** | **Moderator** | | | | | **Composite Moderator M*** |
| --- | --- | --- | --- | --- | --- | --- |
|  | **E1** | **E2** | **E3** | **E4** | **E5** |  |
| No main effect on moderators | 0.06 | 0.058 | 0.06 | 0.049 | 0.057 | 0.055 |
| Some main effect on moderators | 0.058 | 0.048 | 0.068 | 0.062 | 0.045 | 0.056 |

The effect modification rejection rate is the test of the interaction coefficient for each effect moderator ($E_{1}\ldots E_{5},$) and the composite moderator M* (all separately) on the EWTR outcome across 1000 simulated trials (N=1000), set at $\alpha=0.05$. Data generating each moderator is assumed to be independent of each other. Cause-specific hazards model parameters are consistent with the main simulations, with the same treatment effect common across both clinical events. In the scenario with no main effect on the moderators, $\delta_{sj}=0$, and for the scenario with some main effect, parameter values were set to: $\delta_{s1}=0.3, \delta_{s2}=0.02, \delta_{s3}=-0.5, \delta_{s4}=-0.4, \delta_{s5}=0.6$.

Table A4. Empirical Rejection Rate for Effect Modification Under All Scenarios with a Larger Sample Size (N=2000)

| **Scenarios** | **Interaction-to-main effect ratio magnitude (**$\boldsymbol{\Phi}$**)** | **Moderator** | | | | | **Composite Moderator M*** |
| --- | --- | --- | --- | --- | --- | --- | --- |
|  |  | **E1** | **E2** | **E3** | **E4** | **E5** |  |
| **Scenario 1** |  |  |  |  |  |  |  |
| No main effect on moderators | --- | 0.043 | 0.059 | 0.042 | 0.066 | 0.055 | 0.059 |
| Some main effect on moderators | --- | 0.053 | 0.052 | 0.079 | 0.052 | 0.063 | 0.065 |
| **Scenario 2** | 0.5 | 0.242 | 0.057 | 0.054 | 0.132 | 0.07 | 0.133 |
|  | 1 | 0.99 | 0.19 | 0.057 | 0.566 | 0.128 | 0.991 |
|  | 2 | 1.00 | 0.508 | 0.055 | 0.91 | 0.28 | 1.00 |
|  | 4 | 1.00 | 0.712 | 0.045 | 0.986 | 0.40 | 1.00 |
| **Scenario 3** | 0.5 | 0.185 | 0.25 | 0.264 | 0.123 | 0.122 | 0.441 |
|  | 1 | 0.275 | 0.364 | 0.399 | 0.15 | 0.168 | 0.665 |
|  | 2 | 0.313 | 0.439 | 0.488 | 0.189 | 0.186 | 0.812 |
|  | 4 | 0.336 | 0.436 | 0.518 | 0.191 | 0.197 | 0.844 |
| **Scenario 4** | 0.5 | 0.88 | 0.051 | 0.056 | 0.06 | 0.048 | 0.485 |
|  | 1 | 1.00 | 0.193 | 0.097 | 0.072 | 0.059 | 1.00 |
|  | 2 | 1.00 | 0.337 | 0.168 | 0.108 | 0.118 | 1.00 |
|  | 4 | 1.00 | 0.458 | 0.225 | 0.136 | 0.146 | 1.00 |
| **Scenario 5** | 0.5 | 0.191 | 0.232 | 0.306 | 0.153 | 0.206 | 0.627 |
|  | 1 | 0.597 | 0.773 | 0.879 | 0.422 | 0.511 | 0.999 |
|  | 2 | 0.859 | 0.949 | 0.992 | 0.639 | 0.75 | 1.00 |
|  | 4 | 0.933 | 0.982 | 0.999 | 0.742 | 0.845 | 1.00 |

The effect modification rejection rate is the test of the interaction coefficient for each effect moderator ($E_{1}\ldots E_{5},$) and the composite moderator M* (all separately) on the EWTR outcome across 1000 simulated trials (N=2000), set at $\alpha=0.05$. Data generating each moderator is assumed to be independent of each other. Cause-specific hazards model parameters are consistent with the main simulations, with the same treatment effect common across both clinical events

Table A5. Empirical Rejection Rate for Effect Modification Under All Scenarios with Correlated Moderators

| **Scenarios** | **Interaction-to-main effect ratio magnitude (**$\boldsymbol{\Phi}$**)** | **Moderator** | | | | | **Composite Moderator M*** |
| --- | --- | --- | --- | --- | --- | --- | --- |
|  |  | **E1** | **E2** | **E3** | **E4** | **E5** |  |
| **Scenario 1** |  |  |  |  |  |  |  |
| Some main effect on moderators | --- | 0.056 | 0.054 | 0.056 | 0.043 | 0.068 | 0.052 |
| **Scenario 2** | 0.5 | 0.089 | 0.054 | 0.034 | 0.091 | 0.064 | 0.067 |
|  | 1 | 0.735 | 0.115 | 0.057 | 0.215 | 0.099 | 0.697 |
|  | 2 | 0.993 | 0.27 | 0.053 | 0.453 | 0.16 | 0.997 |
|  | 4 | 1.00 | 0.377 | 0.058 | 0.613 | 0.267 | 1.00 |
| **Scenario 3** | 0.5 | 0.098 | 0.087 | 0.096 | 0.074 | 0.04 | 0.134 |
|  | 1 | 0.165 | 0.111 | 0.16 | 0.097 | 0.059 | 0.234 |
|  | 2 | 0.171 | 0.132 | 0.212 | 0.101 | 0.066 | 0.302 |
|  | 4 | 0.186 | 0.168 | 0.192 | 0.117 | 0.069 | 0.331 |
| **Scenario 4** | 0.5 | 0.52 | 0.064 | 0.054 | 0.073 | 0.09 | 0.179 |
|  | 1 | 0.997 | 0.129 | 0.054 | 0.152 | 0.347 | 0.954 |
|  | 2 | 1.00 | 0.23 | 0.048 | 0.255 | 0.668 | 1.00 |
|  | 4 | 1.00 | 0.326 | 0.053 | 0.325 | 0.824 | 1.00 |
| **Scenario 5** | 0.5 | 0.121 | 0.086 | 0.129 | 0.097 | 0.057 | 0.201 |
|  | 1 | 0.384 | 0.249 | 0.45 | 0.221 | 0.104 | 0.872 |
|  | 2 | 0.611 | 0.43 | 0.694 | 0.303 | 0.151 | 0.984 |
|  | 4 | 0.719 | 0.514 | 0.755 | 0.39 | 0.20 | 0.996 |

The effect modification rejection rate is the test of the interaction coefficient for each effect moderator ($E_{1}\ldots E_{5},$) and the composite moderator M* (all separately) on the EWTR outcome across 1000 simulated trials (N=1000), set at $\alpha=0.05$. Data generating each moderator is assumed to be correlated to each other using the covariance structure in **Table 1**. Cause-specific hazards model parameters are consistent with the main simulations, with the same treatment effect common across both clinical events.

Table A6. Empirical Rejection Rate for Effect Modification with Additional Moderators (m=10)

| **Interaction-to-main effect ratio magnitude (**$\boldsymbol{\Phi}$**)** | **Moderator** | | | | | | | | | | **Composite Moderator M*** |
| --- | --- | --- | --- | --- | --- | --- | --- | --- | --- | --- | --- |
|  | **E1** | **E2** | **E3** | **E4** | **E5** | **E6** | **E7** | **E8** | **E9** | **E10** |  |
| 0.5 | 0.052 | 0.071 | 0.06 | 0.056 | 0.068 | 0.068 | 0.103 | 0.067 | 0.08 | 0.12 | 0.101 |
| 1 | 0.097 | 0.081 | 0.116 | 0.093 | 0.064 | 0.184 | 0.308 | 0.113 | 0.198 | 0.222 | 0.50 |
| 2 | 0.122 | 0.156 | 0.154 | 0.081 | 0.102 | 0.34 | 0.508 | 0.196 | 0.28 | 0.351 | 0.84 |
| 4 | 0.129 | 0.158 | 0.212 | 0.103 | 0.115 | 0.42 | 0.573 | 0.224 | 0.313 | 0.443 | 0.932 |

The effect modification rejection rate is the test of the interaction coefficient for each effect moderator ($E_{1}\ldots E_{10},$) and the composite moderator M* (all separately) on the EWTR outcome across 1000 simulated trials (N=1000), set at $\alpha=0.05$. Data generating each moderator is assumed to be independent of each other. Data generating each moderator is assumed to be independent of each other. Cause-specific hazards model parameters are consistent with the main simulations, with the same treatment effect common across both clinical events.

Table A7. Empirical Rejection Rate for Effect Modification Under All Scenarios with the Inclusion of a Null Endpoint

| **Scenarios** | **Interaction-to-main effect ratio magnitude (**$\boldsymbol{\Phi}$**)** | **Moderator** | | | | | **Composite Moderator M*** |
| --- | --- | --- | --- | --- | --- | --- | --- |
|  |  | **E1** | **E2** | **E3** | **E4** | **E5** |  |
| **Scenario 1** |  |  |  |  |  |  |  |
| No main effect on moderators | --- | 0.05 | 0.053 | 0.056 | 0.05 | 0.048 | 0.046 |
| Some main effect on moderators | --- | 0.049 | 0.051 | 0.033 | 0.052 | 0.042 | 0.058 |
| **Scenario 2** | 0.5 | 0.021 | 0.054 | 0.066 | 0.081 | 0.04 | 0.054 |
|  | 1 | 0.049 | 0.037 | 0.037 | 0.065 | 0.054 | 0.054 |
|  | 2 | 0.113 | 0.071 | 0.066 | 0.068 | 0.061 | 0.081 |
|  | 4 | 0.137 | 0.06 | 0.056 | 0.087 | 0.053 | 0.095 |
| **Scenario 3** | 0.5 | 0.054 | 0.044 | 0.049 | 0.055 | 0.042 | 0.058 |
|  | 1 | 0.047 | 0.051 | 0.044 | 0.068 | 0.056 | 0.056 |
|  | 2 | 0.061 | 0.054 | 0.064 | 0.055 | 0.056 | 0.059 |
|  | 4 | 0.055 | 0.055 | 0.046 | 0.052 | 0.046 | 0.047 |
| **Scenario 4** | 0.5 | 0.035 | 0.046 | 0.053 | 0.059 | 0.04 | 0.039 |
|  | 1 | 0.057 | 0.062 | 0.045 | 0.054 | 0.053 | 0.057 |
|  | 2 | 0.123 | 0.056 | 0.061 | 0.055 | 0.054 | 0.076 |
|  | 4 | 0.16 | 0.045 | 0.05 | 0.058 | 0.057 | 0.092 |
| **Scenario 5** | 0.5 | 0.035 | 0.046 | 0.053 | 0.059 | 0.04 | 0.039 |
|  | 1 | 0.057 | 0.062 | 0.045 | 0.054 | 0.053 | 0.057 |
|  | 2 | 0.123 | 0.056 | 0.061 | 0.055 | 0.054 | 0.076 |
|  | 4 | 0.16 | 0.045 | 0.05 | 0.058 | 0.057 | 0.092 |

The effect modification rejection rate is the test of the interaction coefficient for each effect moderator ($E_{1}\ldots E_{5},$) and the composite moderator M* (all separately) on the EWTR outcome across 1000 simulated trials (N=1000), set at $\alpha=0.05$. Data generating each moderator is assumed to be independent of each other. Cause-specific hazards model parameters are consistent with the main simulations, except the treatment effect and treatment-moderator interaction effect is only in the first CSH model.

Table A8. Empirical Rejection Rate for Effect Modification Under All Scenarios with a Single Clinical Endpoint

| **Scenarios** | **Interaction-to-main effect ratio magnitude (**$\boldsymbol{\Phi}$**)** | **Moderator** | | | | | **Composite Moderator M*** |
| --- | --- | --- | --- | --- | --- | --- | --- |
|  |  | **E1** | **E2** | **E3** | **E4** | **E5** |  |
| **Scenario 1** |  |  |  |  |  |  |  |
| No main effect on moderators | --- | 0.055 | 0.047 | 0.036 | 0.051 | 0.057 | 0.036 |
| Some main effect on moderators | --- | 0.064 | 0.049 | 0.106 | 0.041 | 0.046 | 0.061 |
| **Scenario 2** | 0.5 | 0.163 | 0.058 | 0.059 | 0.112 | 0.048 | 0.096 |
|  | 1 | 0.993 | 0.177 | 0.043 | 0.463 | 0.111 | 0.995 |
|  | 2 | 1.00 | 0.542 | 0.049 | 0.874 | 0.24 | 1.00 |
|  | 4 | 1.00 | 0.721 | 0.046 | 0.964 | 0.373 | 1.00 |
| **Scenario 3** | 0.5 | 0.296 | 0.402 | 0.48 | 0.127 | 0.144 | 0.794 |
|  | 1 | 0.38 | 0.48 | 0.546 | 0.174 | 0.181 | 0.868 |
|  | 2 | 0.37 | 0.487 | 0.584 | 0.166 | 0.20 | 0.881 |
|  | 4 | 0.347 | 0.488 | 0.556 | 0.204 | 0.186 | 0.871 |
| **Scenario 4** | 0.5 | 0.911 | 0.081 | 0.067 | 0.051 | 0.045 | 0.554 |
|  | 1 | 1.00 | 0.161 | 0.087 | 0.075 | 0.07 | 1.00 |
|  | 2 | 1.00 | 0.279 | 0.15 | 0.09 | 0.094 | 1.00 |
|  | 4 | 1.00 | 0.373 | 0.193 | 0.13 | 0.108 | 1.00 |
| **Scenario 5** | 0.5 | 0.21 | 0.293 | 0.404 | 0.129 | 0.171 | 0.762 |
|  | 1 | 0.628 | 0.792 | 0.908 | 0.353 | 0.477 | 1.00 |
|  | 2 | 0.838 | 0.942 | 0.993 | 0.552 | 0.676 | 1.00 |
|  | 4 | 0.909 | 0.982 | 0.994 | 0.649 | 0.756 | 1.00 |

The effect modification rejection rate is the test of the interaction coefficient for each effect moderator ($E_{1}\ldots E_{5},$) and the composite moderator M* (all separately) on the EWTR outcome across 1000 simulated trials (N=1000), set at $\alpha=0.05$. Data generating each moderator is assumed to be independent of each other. The cause-specific hazard model (for death) parameters are consistent with the main simulations; only the death clinical event state is considered in the EWTR outcome.

Table A9. Descriptive Characteristics of Subgroups Identified by Composite Moderator M*

| **Decision boundary** |  | **Moderator** | | | |
| --- | --- | --- | --- | --- | --- |
|  | **n** | $\mathbf{E}_{\boldsymbol{1}}$ | $\mathbf{E}_{\boldsymbol{2}}$ | $\mathbf{E}_{\boldsymbol{3}}$ | $\mathbf{E}_{\boldsymbol{4}}$ |
|  |  | **Age (stand. yrs)** | **CAD n (%)** | **Hypertension n (%)** | **CHF n (%)** |
|  |  | $w_{1}=-27.9$ | $w_{2}=-35.3$ | $w_{2}=-44.1$ | $w_{4}=48.3$ |
| $M$*$\geq24.2$ | 735 | -1.12 (0.99) | 101 (13.7) | 357 (48.6) | 337 (45.9) |
| $M$*$<24.2$ | 3325 | 0.25 (0.82) | 1450 (43.6) | 2519 (75.8) | 602 (18.1) |

CHF=congestive heart failure; CAD=coronary artery disease. Moderators are standardized (mean=0, SD=1 or coded -1/2, 1/2). Median weights reported across 5-folds. $w_{1}\ldots w_{4}$ represent the estimated weights. Regression lines for rate- and rhythm-control cross at the vertical dashed line ($M_{intersection}^{*}=24.2$), representing the decision boundary for treatment recommendation. Participants with M* values to the left are recommended rate-control, whereas those to the right are recommended rhythm-control.

Table A10. Descriptive Characteristics of Clusters Identified by Composite Moderator M* using Gaussian-Mixture Model

| **Descriptive characteristics** | **Cluster 1 (n=534)** | **Cluster 2 (n=3290)** | **Cluster 3 (n=236)** |
| --- | --- | --- | --- |
| Composite moderator M*, mean ± SD | -93.9 ± 17 | -13.2 ± 32.2 | 83.7 ± 24.8 |
| Age, standardized years, mean ± SD | 0.84 ± 0.44 | -0.02 ± 0.93 | -1.59 ± 0.86 |
| CHF, n (%) | 10 (1.9) | 766 (23.3) | 163 (69.1) |
| Hypertension, n (%) | 519 (97.2) | 2267 (68.9) | 90 (38.1) |
| CAD, n (%) | 381 (71.3) | 1130 (34.3) | 40 (16.9) |
| Posterior probability, mean ± SD | 0.62 ± 0.09 | 0.70 ± 0.08 | 0.69 ± 0.15 |

CHF=congestive heart failure; CAD=coronary artery disease. Composite moderator M* derived from 5-fold cross-validation. Gaussian-mixture model assumes distribution of M* is finite mixture of normal and fits the model by maximum likelihood with the EM algorithm. It estimates a posterior probability that each subject belongs to each cluster. Three clusters were prespecified to achieve one cluster of preference for each treatment and one cluster of uncertainty: cluster 1 (blue) is participant profile recommended rate-control, cluster 3 (pink) is participant profile recommended for rhythm-control, and cluster 2 is participant profile at the decision boundary.
